# Development of Sendai Virus Vectors and their Potential Applications in Gene Therapy and Regenerative Medicine

**DOI:** 10.2174/156652312802762518

**Published:** 2012-10

**Authors:** Mahito Nakanishi, Makoto Otsu

**Affiliations:** 1 Research Center for Stem Cell Engineering, National Institute of Advanced Industrial Science and Technology (AIST), Tsukuba, Ibaraki, Japan; 2 Division of Stem Cell Therapy, Center for Stem Cell Biology and Regenerative Medicine, The Institute of Medical Science, The University of Tokyo, Tokyo, Japan; 3 Stem Cell Bank, Center for Stem Cell Biology and Regenerative Medicine, The Institute of Medical Science, The University of Tokyo, Tokyo, Japan

**Keywords:** Sendai virus, gene therapy, nuclear reprogramming, induced pluripotent stem cells (iPSCs).

## Abstract

Gene delivery/expression vectors have been used as fundamental technologies in gene therapy since the 1980s. These technologies are also being applied in regenerative medicine as tools to reprogram cell genomes to a pluripotent state and to other cell lineages. Rapid progress in these new research areas and expectations for their translation into clinical applications have facilitated the development of more sophisticated gene delivery/expression technologies. Since its isolation in 1953 in Japan, Sendai virus (SeV) has been widely used as a research tool in cell biology and in industry, but the application of SeV as a recombinant viral vector has been investigated only recently. Recombinant SeV vectors have various unique characteristics, such as low pathogenicity, powerful capacity for gene expression and a wide host range. In addition, the cytoplasmic gene expression mediated by this vector is advantageous for applications, in that chromosomal integration of exogenous genes can be undesirable. In this review, we introduce a brief historical background on the development of recombinant SeV vectors and describe their current applications in gene therapy. We also describe the application of SeV vectors in advanced nuclear reprogramming and introduce a defective and persistent SeV vector (SeVdp) optimized for such reprogramming.

## INTRODUCTION

Since the finding in the 1970s that cultured cells can take up nucleic acids with the aid of cationic molecules, techniques of gene delivery and expression in mammalian cells have been used widely in modern biology. Development of sophisticated gene delivery tools in the 1980s such as retroviral vectors [1, 2], adenoviral vectors [3] and cationic lipid-based reagents [4] facilitated the translation of these technologies to human gene therapy. Prototypes of the current gene delivery tools used in research and clinics were mostly established in those early periods, and then followed by significant progress in each technology [5-8].

Although various formulations of DNA–carrier complexes have been developed, recombinant viral vectors are still used as the primary choice for delivering therapeutic genes because of their efficacy. Nonetheless, the refinement of current viral vectors and development of novel viral vectors are still desired, as none of the current viral vectors satisfies all of the requirements for various applications [9, 10]. In principle, any animal virus could be tailored to form gene delivery vectors provided it can accept exogenous genes as a part of their viral genome. However, their utility is limited by various factors, including pathogenicity to humans and the availability of procedures for large-scale production. Even if candidate viruses satisfy these minimum requirements, they should have clear advantages over “classical” viral vectors in being established as practical tools. The Sendai virus (SeV) vector is a newcomer in the field with unique characteristics, making it distinct from other viral vectors.

In this review, we introduce characteristics of various SeV vectors, a unique RNA virus-based gene delivery/expression system and describe recent progress in the application of SeV vectors to molecular therapy and to advanced nuclear reprogramming.

## DEVELOPMENT OF RECOMBINANT SENDAI VIRUS VECTORS

SeV (mouse parainfluenza virus type 1, hemagglutinating virus of Japan (HVJ)) is a nonsegmented negative-strand RNA virus belonging to the *Paramyxovirus* family [11] with a large spherical shape and an average diameter of 260 nm [12]. A SeV virion consists of the nucleocapsid (genomic RNA complexed with NP, P and L proteins), an envelope (a lipid bilayer with F and HN proteins) and a matrix (M protein) connecting the nucleocapsid and envelope (Fig. (**1**)).

Since its first isolation in the 1950s in Japan [13], SeV has occupied a unique position as a research tool for basic and applied biology. Long before being characterized at a molecular level, SeV particles inactivated by brief exposure to ultraviolet light or by treatment with alkylation reagents have been widely used as research tools as a fusogenic agent to make hybrid cells [14] and as a tool for delivering macromolecules (protein and nucleic acid) into mammalian cells through membrane fusion [15, 16]. Intact live SeV was also used in the large-scale production of interferon (IFN) as an inducer of IFN expression [17, 18]. This is partly because SeV is neither tumorigenic nor pathogenic to humans and because SeV-mediated membrane fusion occurs efficiently with exceptionally low species and cell specificity. In addition, chicken egg-adapted SeV is readily propagated on the large scale: as much as one milligram (as protein) of purified SeV can be recovered from a single fertilized egg (Fig. (**1**)). All of these characteristics have made SeV a preferred research tool over other envelope viruses.

The complete genome sequence of the SeV Z strain was determined in 1986: first among the paramyxoviruses [19]. SeV has a 15,384-nucleotide single-strand RNA genome, consisting of six cistrons (Fig. (**2**)). Each cistron has concise transcription initiation and termination signals and is transcribed to mRNA encoding a single polypeptide (except for the P cistron encoding P, C and V proteins; Fig. (**2**)). This simple genome structure encouraged us to develop a recombinant SeV vector by replacing the genes dispensable for gene expression (F, HN and M genes) with therapeutic genes. However, this idea was hampered by the lack of methodology to modify the viral genome, as SeV has no DNA intermediate in its replication cycle.

Reconstitution of recombinant SeV from full-length genomic cDNA was accomplished in 1995 [20] following the establishment of a breakthrough strategy allowing the reconstitution of Rabies virus from cDNA in 1994 [21]. Since then, various SeV vectors installed with exogenous genes have been generated based on the wild-type SeV strain. In the first generation of SeV vectors, exogenous cDNA was installed between the 3′ terminus and the NP gene of a full-length SeV genome [22]. These SeV vectors were replication competent and could produce large amounts of exogenous gene products when cultured in fertilized chicken eggs [23]. For medical and other practical applications, replication-defective SeV vectors with deletion in the F gene were developed subsequently [24]. These vectors were shown to induce transient but very strong gene expression so their application as tools for gene therapy and vaccine has been explored, as described below.

## APPLICATION OF SEV VECTORS IN MOLECULAR THERAPY

At the early stage of SeV vector development, the feasibility of applying SeV vectors to various cell types was examined extensively *in vitro* and *in vivo*. SeV naturally replicates in respiratory epithelial cells and is a major pathogen causing respiratory symptoms in mice. In addition to the lung/airway epithelium [25], a recombinant SeV vector can induce strong *ex*-gene expression in the cardiovascular system [26], in retinal epithelium [27], in hepatocytes [12], in colonic epithelium [28], in neurons [24], in dendritic cells [29] and in human hematopoietic stem cells [30]. This remarkably wide host range partly depends on the fact that the primary SeV receptor, sialic acid, is distributed universally among animal cells; the presence of a ubiquitous secondary receptor indispensable for SeV-mediated membrane fusion has also been suggested [31]. In addition, SeV vectors rely for their gene expression only on virus-encoded RNA polymerase and tubulin, a ubiquitous conserved cytoskeletal protein [32].

The application of SeV vectors in molecular medicine is dependent on, and is on some occasions restricted by, powerful but transient gene expression, wide host cell specificity, low pathogenicity and strong immunogenicity. To date, the feasibility for using SeV vectors clinically has been examined in the following areas: 1) as a live attenuated vaccine; 2) in gene therapy for critical limb ischemia; and 3) in cancer gene therapy.

Recombinant SeV vectors have been most intensively investigated as a vaccine platform for inducing mucosal immunity [33]. SeV was originally investigated as a xenotropic live-attenuated vaccine as it was known to have antigenicity shared with human parainfluenza virus type 1 (hPIV-1), an important human pathogen causing pneumonia and laryngotracheobronchitis. Results of a phase 1 trial where live wild-type SeV was administered intranasally showed that SeV induced anti-hPIV-1 immunity effectively without any severe adverse events [34]. This result further emphasized the nonpathogenic nature of SeV to humans, partly because of the sensitivity of SeV replication to IFN [35]. Subsequently, replication-competent recombinant SeV vectors expressing envelope proteins of hPIV-1, hPIV-2, hPIV-3 and respiratory syncytial virus (RSV) were developed and their effectiveness was proven in model animals [36]. SeV vectors have also been investigated as platforms for vaccines against the human immunodeficiency virus [37] and influenza viruses [38].

Replication-defective (F-defective) SeV vectors expressing the angiogenic cytokine fibroblast growth factor-2 (FGF2) have been developed for treatment of critical limb ischemia [39, 40]. A phase 1/2a clinical trial using the SeV-FGF2 vector was performed in 2006 at Kyushu University (Fukuoka, Japan) and up to 5 × 10^9^ plaque-forming units (pfu)/60 kg (body weight) of rSeV-FGF2 were administrated intramuscularly. Although the outcomes for patients remain unpublished, no severe adverse events were reported. This trial was the first to administer a recombinant SeV vector to humans directly by injection.

Applications of SeV vectors to cancer gene therapy have been investigated at the preclinical stage. In addition to the stimulation of dendritic cells with SeV vector expressing IFNβ (rSeV-IFNβ) [29], virotherapy with the unique host-restricted SeV vector rSeV/dMFct14(uPA2) (“BioKnife”) has been developed [41]. Infectivity of wild-type SeV absolutely requires the cleavage of the precursor F_0_ protein to F_1_ and F_2_ subunits [42], resulting in exposure of the hydrophobic N-terminus of the F_1_ subunit [43]. Serine protease is responsible for this cleavage in the lungs of natural hosts [44]. BioKnife is created by altering the structure of this cleavage site to that optimal to urokinase-type plasminogen activator (uPA) and by deletion of the *M* gene to interfere with virion production [41]. These modifications restrict the spread of this uPA-dependent recombinant SeV by membrane fusion between cells. As uPA is often activated on cancer cell surfaces and is responsible for metastasis, this virus spreads preferentially to metastatic tumors and destroys them by its intrinsic cytotoxic activity. BioKnife has been reported to be effective in treating intractable cancers such as malignant glioblastomas [45] and malignant pleural mesotheliomas [46] in animal models.

## NUCLEAR REPROGRAMMING WITH SEV VECTORS

Gene delivery/expression technologies are also indispensable for reprogramming somatic cell genomes by the ectopic expression of transcription factors. The concept of nuclear reprogramming was first proposed in the 1970s, based on experiments with somatic cell fusion and was proven by the discovery of *MyoD*, a gene encoding a master transcription factor inducing the dynamic transition of fibroblasts to myoblasts [47]. However, no other genes encoding a master transcription factor capable of reprogramming by itself have been identified.

Reprogramming skin fibroblasts into induced pluripotent stem cells (iPSCs) by the ectopic expression of four reprogramming factors (Oct4, Sox2, Klf4 and c-Myc) opened a new era in this field [48-51]. Human iPSCs have a capacity to differentiate to all three germ layers, as do embryonic stem cells (ESCs), while tissue cells generated from iPSCs can escape from immunological rejection when transplanted to the host. In addition, using an iPSC line can avoid the ethical concern that generation of ESCs requires the destruction of normal human embryos. All of these features likely make human iPSC lines ideal sources for gene and cell therapy.

In addition to these practical aspects, the discovery of iPSCs also introduced the novel concept that cell fate conversion needs the cooperation of multiple gene products in a single cell. As genomic reprogramming is a relatively slow process, expression of the exogenous transcription factors has to be sustained for 10–20 days [52]. On the other hand, these exogenous genes should be irreversibly suppressed or—ideally—should be removed from the reprogrammed cells. This latter point is important both for avoiding undesired side effects (cell transformation and insertional mutagenesis) and for maintaining full pluripotency in these cells [53].

Following the initial successes in nuclear reprogramming using gamma-retrovirus or lentivirus vectors, both of which cause provirus insertion into host genomes, researchers have made substantial efforts to establish methods for generating *ex*-gene-free iPSCs [53]. However, it is difficult to satisfy controversial requirements described above thoroughly with conventional gene delivery/expression tools. These and other necessities have directed the attention of researchers to the gene delivery/expression technologies once again and should facilitate the development of novel technology best suited for genomic reprogramming.

The SeV vector stands unique among other vector systems in genomic reprogramming because it can express the reprogramming genes without chromosomal integration. Wild-type SeV vectors installed with *Oct4*, *Sox2*, *Klf4* and *c-Myc* cDNA were reported to generate *ex*-gene-free iPSCs, dependent on passive elimination of the genome through cell passage [54]. This prototype was replaced with a less cytotoxic backbone [55] and is now available commercially. The SeV vector with a temperature-sensitive (*ts*) mutation was also reported to facilitate the erasure of the vector genome [56]. However, as each of the reprogramming genes is installed on separate vectors, the balance of their expression levels is likely to vary among infected cells (Fig. (**3**)). Therefore, the biological characteristics of each iPSC line generated by these separate SeV vectors should be examined carefully, as the balance of expression of reprogramming genes might affect the quality of the iPSC line [57].

Defective and persistent Sendai virus (SeVdp) vectors are now also recognized as a superior tool for iPSC generation thanks to their remarkably high potential and simplicity [30]. The SeVdp vector was developed by one of us (MN) based on noncytotoxic *ts* mutant SeV strain clone 151 (SeV cl.151), originally isolated in 1979 [58]. Distinct from wild-type cytotoxic SeV and from other *ts* mutant SeV strains with defects in gene expression, SeV cl.151 is unique because it readily establishes stable persistent infection in cultured cells at 37 °C and strong expression of viral genes can be sustained indefinitely [31]. We identified the mutations responsible for this phenotype [30, 59, 60] and showed that this virus escapes from the intrinsic cytotoxicity of SeV by a defect in the induction of IFNβ production in target cells [60]. This phenotype is unique among cytoplasmic RNA viruses and is ideal for stable and reproducible gene expression.

An SeVdp vector suitable for generating *ex*-gene-free iPSCs (SeVdp-iPSCs) was created by deleting the *M*, *F* and *HN* genes dispensable for persistent gene expression and by installing four reprogramming genes instead (Fig. (**4**)). This “all-in-one” genome structure is essential both for certifying simultaneous expression of all the reprogramming genes at a constant ratio in a single cell (Fig. (**3**)), and for preventing secondary virion production from infected cells [30]. After completion of reprogramming, the SeVdp-iPS genome can be erased through suppression of viral L protein expression with short interfering RNA (siRNA) [30]. The human iPSC lines generated are *ex*-gene free and are quite uniform in their characteristics: more than 80% of the colonies are positive for TRA-1-60, a reliable marker for pluripotency [61] (Nishimura and Nakanishi, unpublished).

A broad target cell range is another important characteristic of SeV vector-mediated nuclear reprogramming. In addition to skin fibroblasts, SeV vectors can reprogram the nuclei of various human cells, including CD34^+^ cord blood cells [62], activated T lymphocytes [55] and monocytes (Nishimura and Nakanishi, unpublished). Generation of iPSC lines from peripheral blood cells, especially from nondividing monocytes, will be quite important for the practical use of tailor-made iPSCs in regenerative and molecular medicine.

## FUTURE PERSPECTIVES

As we show in this review, recombinant SeV vectors are powerful tools in basic research, in molecular therapy and in regenerative medicine. Among various applications of SeV vectors, generation of human iPSCs by nuclear reprogramming is attracting broad interest and is highly valued. Although SeV vectors can generate *ex*-gene-free iPSCs quite efficiently compared with other gene delivery/expression systems, claims of total superiority should be treated cautiously, as “*ex*-gene free” is highly desirable but might not be sufficient for clinical-grade iPSC lines [53]. The long-term stability of genome structure and epigenetic conditions should be examined before the clinical application of iPSC lines generated using SeV vectors.

Since the discovery that the genomes of somatic cells could be reprogrammed to generate pluripotent iPSCs, the notion of direct genomic reprogramming of somatic cells to other cell lineages (either to terminally differentiated cells or to tissue stem cells) has also attracted significant interest [63]. Direct reprogramming (*trans*-differentiation) relies on the same strategy for iPSC generation, that is, ectopic expression of multiple lineage-specific protein factors in a single cell followed with culture under defined conditions optimized for each target cell type. This procedure has been investigated not only in cells cultured *in vitro* but also in disease target tissues *in situ *[64, 65]. Direct genomic reprogramming can eliminate the potential risk of tumorigenicity in iPSC-mediated tissue cells, unless the cellular life span is reset to be infinite. This approach might also accelerate the generation of target tissue cells through bypassing the time-consuming process of iPSC generation.

Currently, direct genomic reprogramming is investigated mostly by using classical retro/lentivirus vectors. However, low reprogramming efficiency and chromosomal integration of exogenous reprogramming genes limit their translation into clinical applications. These obstacles could be overcome with the use of SeV vectors. As described above, these have the potential to deliver exogenous genes into target tissues *in situ*. The development of less antigenic, “smart” SeV vectors equipped with the machinery for controlling gene expression and genome stability *in vivo* should contribute to translation of the current direct genomic reprogramming technologies into clinical applications. Further refinement of SeV vectors through basic research is highly desired and will bring a promising future to the fields of gene therapy and regenerative medicine.

## Figures and Tables

**Fig. (1):**
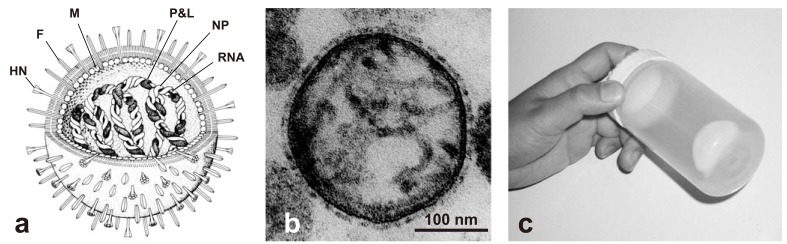
Characteristics of Sendai virus. (**a**) Schematic structure of Sendai virus. Reprinted with the permission of Nikkei Science, Inc. (**b**) Cross-section view of Sendai virus examined with transmission electron microscopy (courtesy of Dr. Takao Senda, Fujita Health University School of Medicine). (**c**) Purified Sendai virus in a centrifuge tube. Sendai virus was propagated in 500 fertilized chicken eggs and was purified extensively by sucrose step centrifugation. The large off-white pellet contains about 500 mg of purified Sendai virus as protein.

**Fig. (2):**
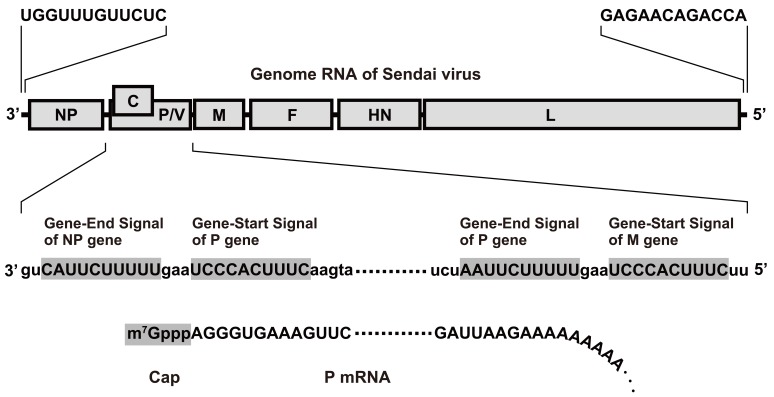
Genome structure of the Sendai virus. Transcription of capped mRNAs starts from the transcription initiation signal with RNA-dependent RNA polymerase (L protein). Transcription ends at the transcription termination signal, followed by a poly-A signal.

**Fig. (3):**
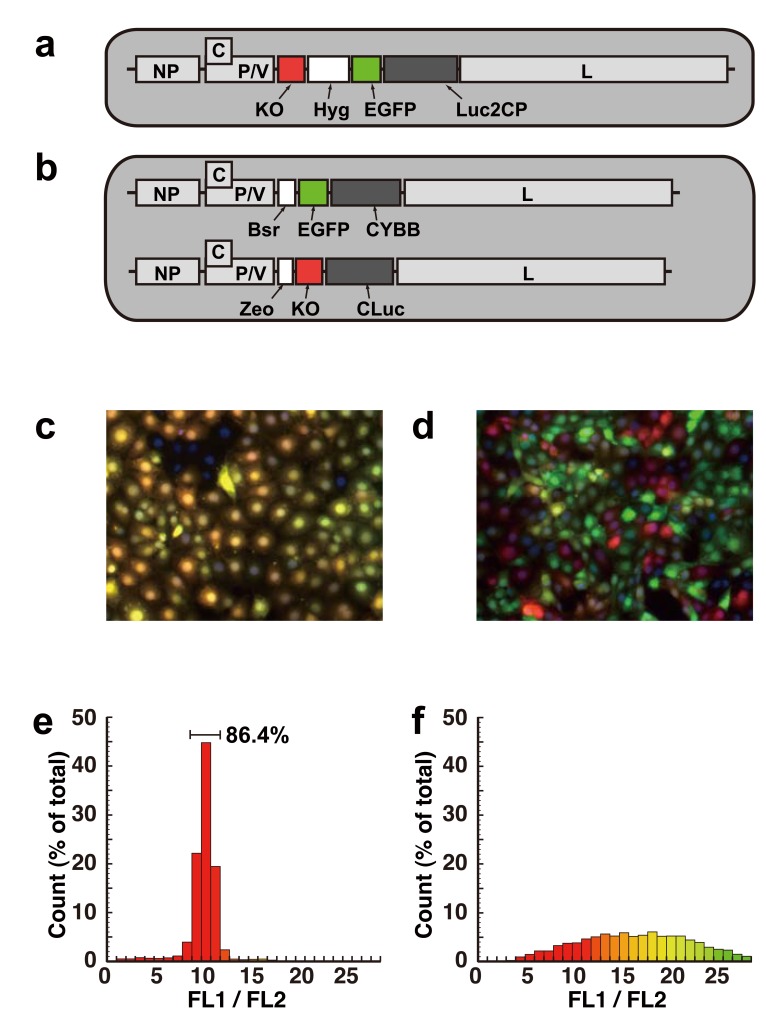
Compatibility of two independent SeVdp vectors in a single cell. (**Top**) Structure of SeV vectors. cDNA sequences encoding for enhanced green fluorescent protein (EGFP) cDNA and Kusabira orange (KO) were installed on a single SeV vector SeVdp(KO/EGFP) (**a**) or on two SeV vectors SeVdp(KO) and SeVdp(EGFP) separately (**b**). (Middle) Fluorescence images of cells expressing KO and EGFP from these SeV vectors. Fluorescence microscopy images of KO and of EGFP were obtained separately with specific filter sets and merged after being converted to an artificial color output (green for EGFP and red for KO). Cells carry a single SeV vector SeVdp(KO/EGFP) (**c**) or mixture of two SeV vectors SeVdp(KO) and SeVdp(EGFP) (**d**). (Bottom) Expression levels of KO and EGFP were analyzed quantitatively using flow cytometry. The ratio of the signal intensities of EGFP and KO in each cell is shown as a histogram. Cells carry a single SeV vector SeVdp(KO/EGFP) (**e**) or mixture of two SeV vectors SeVdp(KO) and SeVdp(EGFP) (**f**). Reprinted from reference 26 with permission.

**Fig. (4):**
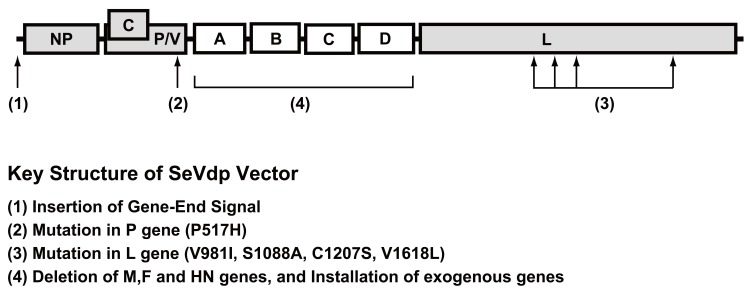
Genome structure of defective and persistent Sendai virus (SeVdp) vector. SeVdp has mutations in the *L* and *P* genes, which are responsible for low cytotoxicity and for defective induction of IFNβ. The *M*, *F* and *HN* genes are deleted and replaced with genes of interest (A–D). SeVdp-iPS was installed with *Oct4*, *Sox2*, *Klf4* and *c-Myc* cDNAs on a single vector.

## References

[1] Shimotohno K, Temin HM (1981). Formation of infectious progeny virus after insertion of herpes simplex thymidine kinase gene into DNA of an avian retrovirus. Cell.

[2] Wei CM, Gibson M, Spear PG, Scolnick EM (1981). Construction and isolation of a transmissible retrovirus containing the src gene of Harvey murine sarcoma virus and the thymidine kinase gene of herpes simplex virus type 1. J Virol.

[3] Berkner KL, Sharp PA (1984). Expression of dihydrofolate reductase and of the adjacent EIb region in an Ad5-dihydrofolate reductase recombinant virus. Nucleic Acids Res.

[4] Felgner PL, Gadek TR, Holm M (1987). Lipofection: a highly efficient lipid-mediated DNA-transfection procedure. Proc Natl Acad Sci U S A.

[5] Khare R, Chen CY, Weaver EA, Barry MA (2011). Advances and future challenges in adenoviral vector pharmacology and targeting. Curr Gene Ther.

[6] Tiera MJ, Shi Q, Winnik FM, Fernandes JC (2011). Polycation-based gene therapy: current knowledge and new perspectives. Curr Gene Ther.

[7] Yi Y, Noh MJ, Lee KH (2011). Current advances in retroviral gene therapy. Curr Gene Ther.

[8] Kumar P, Woon-Khiong C (2011). Optimization of lentiviral vectors generation for biomedical and clinical research purposes: contemporary trends in technology development and applications. Curr Gene Ther.

[9] Jang JH, Schaffer DV, Shea LD (2011). Engineering biomaterial systems to enhance viral vector gene delivery. Mol Ther.

[10] Warnock JN, Daigre C, Al-Rubeai M (2011). Introduction to viral vectors. Methods Mol Biol.

[11] Lamb RA, Kolakofsky D (2001). Paramyxoviridae: The Viruses and Their Replication. Fundamental Virology Fourth Edition.

[12] Fujita S, Eguchi A, Okabe J (2006). Sendai virus-mediated gene delivery into hepatocytes via isolated hepatic perfusion. Biol Pharm Bull.

[13] Kuroya M, Ishida N (1953). Newborn virus pneumonitis (type Sendai) II The isolation of a new virus possessing hemagglutinin activity. Yokohama Med Bull.

[14] Okada Y (1993). Sendai virus-induced cell fusion. Methods Enzymol.

[15] Kato K, Nakanishi M, Kaneda Y, Uchida T, Okada Y (1991). Expression of hepatitis B virus surface antigen in adult rat liver. Co-introduction of DNA and nuclear protein by a simplified liposome method. J Biol Chem.

[16] Uchida T, Kim J, Yamaizumi M, Miyake Y, Okada Y (1979). Reconstitution of lipid vesicles associated with HVJ (Sendai virus) sikes. Purification and some properties of vesicles containing nontoxic fragment A of diphtheria toxin. J Cell Biol.

[17] Johnston MD (1981). The characteristics required for a Sendai virus preparation to induce high levels of interferon in human lymphoblastoid cells. J Gen Virol.

[18] Johnston MD, Fantes KH, Finter NB (1978). Factors influencing production of interferon by human lymphoblastoid cells. Adv Exp Med Biol.

[19] Shioda T, Iwasaki K, Shibuta H (1986). Determination of the complete nucleotide sequence of the Sendai virus genome RNA and the predicted amino acid sequences of the F, HN and L proteins. Nucleic Acids Res.

[20] Garcin D, Pelet T, Calain P (1995). A highly recombinogenic system for the recovery of infectious Sendai paramyxovirus from cDNA: generation of a novel copy-back nondefective interfering virus. EMBO J.

[21] Schnell MJ, Mebatsion T, Conzelmann KK (1994). Infectious rabies viruses from cloned cDNA. EMBO J.

[22] Hasan MK, Kato A, Shioda T (1997). Creation of an infectious recombinant Sendai virus expressing the firefly luciferase gene from the 3' proximal first locus. J Gen Virol.

[23] Moriya C, Shioda T, Tashiro K (1998). Large quantity production with extreme convenience of human SDF-1alpha and SDF-1beta by a Sendai virus vector. FEBS Lett.

[24] Li HO, Zhu YF, Asakawa M (2000). A cytoplasmic RNA vector derived from nontransmissible Sendai virus with efficient gene transfer and expression. J Virol.

[25] Yonemitsu Y, Kitson C, Ferrari S (2000). Efficient gene transfer to airway epithelium using recombinant Sendai virus. Nat Biotechnol.

[26] Masaki I, Yonemitsu Y, Komori K (2001). Recombinant Sendai virus-mediated gene transfer to vasculature: a new class of efficient gene transfer vector to the vascular system. FASEB J.

[27] Murakami Y, Ikeda Y, Yonemitsu Y (2008). Newly-developed Sendai virus vector for retinal gene transfer: reduction of innate immune response via deletion of all envelope-related genes. J Gene Med.

[28] Goto T, Morishita M, Nishimura K (2006). Novel mucosal insulin delivery systems based on fusogenic liposomes. Pharm Res.

[29] Shibata S, Okano S, Yonemitsu Y (2006). Induction of efficient antitumor immunity using dendritic cells activated by recombinant Sendai virus and its modulation by exogenous IFN-beta gene. J Immunol.

[30] Nishimura K, Sano M, Ohtaka M (2011). Development of defective and persistent Sendai virus vector: a unique gene delivery/expression system ideal for cell reprogramming. J Biol Chem.

[31] Eguchi A, Kondoh T, Kosaka H (2000). Identification and characterization of cell lines with a defect in a post-adsorption stage of Sendai virus-mediated membrane fusion. J Biol Chem.

[32] Mizumoto K, Muroya K, Takagi T (1995). Protein factors required for in vitro transcription of Sendai virus genome. J Biochem.

[33] Hurwitz JL (2008). Development of recombinant Sendai virus vaccines for prevention of human parainfluenza and respiratory syncytial virus infections. Pediatr Infect Dis J.

[34] Slobod KS, Shenep JL, Lujan-Zilbermann J (2004). Safety and immunogenicity of intranasal murine parainfluenza virus type 1 (Sendai virus) in healthy human adults. Vaccine.

[35] Bousse T, Chambers RL, Scroggs RA, Portner A, Takimoto T (2006). Human parainfluenza virus type 1 but not Sendai virus replicates in human respiratory cells despite IFN treatment. Virus Res.

[36] Jones B, Zhan X, Mishin V (2009). Human PIV-2 recombinant Sendai virus (rSeV) elicits durable immunity and combines with two additional rSeVs to protect against hPIV-1, hPIV-2, hPIV-3, and RSV. Vaccine.

[37] Yu S, Feng X, Shu T (2008). Potent specific immune responses induced by prime-boost-boost strategies based on DNA adenovirus and Sendai virus vectors expressing gag gene of Chinese HIV-1 subtype B. Vaccine.

[38] Le TV, Mironova E, Garcin D, Compans RW (2011). Induction of influenza-specific mucosal immunity by an attenuated recombinant Sendai virus. PLoS One.

[39] Masaki I, Yonemitsu Y, Yamashita A (2002). Angiogenic gene therapy for experimental critical limb ischemia: acceleration of limb loss by overexpression of vascular endothelial growth factor 165 but not of fibroblast growth factor-2. Circ Res.

[40] Onimaru M, Yonemitsu Y, Tanii M (2002). Fibroblast growth factor-2 gene transfer can stimulate hepatocyte growth factor expression irrespective of hypoxia-mediated downregulation in ischemic limbs. Circ Res.

[41] Kinoh H, Inoue M, Komaru A (2009). Generation of optimized and urokinase-targeted oncolytic Sendai virus vectors applicable for various human malignancies. Gene Ther.

[42] Homma M, Ouchi M (1973). Trypsin action on the growth of Sendai virus in tissue culture cells 3 Structural difference of Sendai viruses grown in eggs and tissue culture cells. J Virol.

[43] Gething MJ, White JM, Waterfield MD (1978). Purification of the fusion protein of Sendai virus: analysis of the NH2- terminal sequence generated during precursor activation. Proc Natl Acad Sci U S A.

[44] Kido H, Yokogoshi Y, Sakai K (1992). Isolation and characterization
of a novel trypsin-like protease found in rat bronchiolar epithelial
Clara cells. A possible activator of the viral fusion glycoprotein. J Biol Chem.

[45] Hasegawa Y, Kinoh H, Iwadate Y (2010). Urokinase-targeted fusion by oncolytic Sendai virus eradicates orthotopic glioblastomas by pronounced synergy with interferon-beta gene. Mol Ther.

[46] Morodomi Y, Yano T, Kinoh H (2012). BioKnife a uPA activity-dependent oncolytic Sendai virus eliminates pleural spread of malignant mesothelioma via simultaneous stimulation of uPA expression. Mol Ther.

[47] Davis RL, Weintraub H, Lassar AB (1987). Expression of a single transfected cDNA converts fibroblasts to myoblasts. Cell.

[48] Okita K, Ichisaka T, Yamanaka S (2007). Generation of germline-competent induced pluripotent stem cells. Nature.

[49] Takahashi K, Tanabe K, Ohnuki M (2007). Induction of pluripotent stem cells from adult human fibroblasts by defined factors. Cell.

[50] Takahashi K, Yamanaka S (2006). Induction of pluripotent stem cells from mouse embryonic and adult fibroblast cultures by defined factors. Cell.

[51] Yu J, Vodyanik MA, Smuga-Otto K (2007). Induced pluripotent stem cell lines derived from human somatic cells. Science.

[52] Jaenisch R, Young R (2008). Stem cells the molecular circuitry of pluripotency and nuclear reprogramming. Cell.

[53] Gonzalez F, Boue S, Izpisua Belmonte JC (2011). Methods for making induced pluripotent stem cells: reprogramming a la carte. Nat Rev Genet.

[54] Fusaki N, Ban H, Nishiyama A, Saeki K, Hasegawa M (2009). Efficient induction of transgene-free human pluripotent stem cells using a vector based on Sendai virus, an RNA virus that does not integrate into the host genome. Proc Jpn Acad Ser B Phys Biol Sci.

[55] Seki T, Yuasa S, Oda M (2010). Generation of induced pluripotent stem cells from human terminally differentiated circulating T cells. Cell Stem Cell.

[56] Ban H, Nishishita N, Fusaki N (2011). Efficient generation of transgene-free human induced pluripotent stem cells (iPSCs) by temperature-sensitive Sendai virus vectors. Proc Natl Acad Sci U S A.

[57] Carey BW, Markoulaki S, Hanna JH (2011). Reprogramming factor stoichiometry influences the epigenetic state and biological properties of induced pluripotent stem cells. Cell Stem Cell.

[58] Yoshida T, Nagai Y, Maeno K (1979). Studies on the role of M protein in virus assembly using a ts mutant of HVJ (Sendai virus). Virology.

[59] Kondo T, Yoshida T, Miura N, Nakanishi M (1993). Temperature-sensitive phenotype of a mutant Sendai virus strain is caused by its insufficient accumulation of the M protein. J Biol Chem.

[60] Nishimura K, Segawa H, Goto T (2007). Persistent and stable gene expression by a cytoplasmic RNA replicon based on a noncytopathic variant Sendai virus. J Biol Chem.

[61] Chan EM, Ratanasirintrawoot S, Park IH (2009). Live cell imaging distinguishes bona fide human iPS cells from partially reprogrammed cells. Nat Biotechnol.

[62] Takayama N, Nishimura S, Nakamura S (2010). Transient activation of c-MYC expression is critical for efficient platelet generation from human induced pluripotent stem cells. J Exp Med.

[63] Vierbuchen T, Wernig M (2011). Direct lineage conversions: unnatural but useful?. Nat Biotechnol.

[64] Qian L, Huang Y, Spencer CI (2012). In vivo reprogramming of murine cardiac fibroblasts into induced cardiomyocytes. Nature.

[65] Song K, Nam YJ, Luo X (2012). Heart repair by reprogramming non-myocytes with cardiac transcription factors. Nature.

